# Anti‐tumor effects of a nonsteroidal anti‐inflammatory drug zaltoprofen on chondrosarcoma via activating peroxisome proliferator‐activated receptor gamma and suppressing matrix metalloproteinase‐2 expression

**DOI:** 10.1002/cam4.1438

**Published:** 2018-03-23

**Authors:** Takashi Higuchi, Akihiko Takeuchi, Seiichi Munesue, Norio Yamamoto, Katsuhiro Hayashi, Hiroaki Kimura, Shinji Miwa, Hiroyuki Inatani, Shingo Shimozaki, Takashi Kato, Yu Aoki, Kensaku Abe, Yuta Taniguchi, Hisaki Aiba, Hideki Murakami, Ai Harashima, Yasuhiko Yamamoto, Hiroyuki Tsuchiya

**Affiliations:** ^1^ Department of Orthopaedic Surgery Kanazawa University Graduate School of Medical Sciences 13‐1 Takara‐machi Kanazawa 920‐8641 Japan; ^2^ Department of Biochemistry and Molecular Vascular Biology Kanazawa University Graduate School of Medical Sciences 13‐1 Takara‐machi Kanazawa 920‐8640 Japan

**Keywords:** Chondrosarcoma, MMP2, nonsteroidal anti‐inflammatory drugs, PPAR*γ*, zaltoprofen

## Abstract

Surgical resection is the only treatment for chondrosarcomas, because of their resistance to chemotherapy and radiotherapy; therefore, additional strategies are crucial to treat chondrosarcomas. Peroxisome proliferator‐activated receptor gamma (PPAR *γ*) is a ligand‐activated transcription factor, which has been reported as a possible therapeutic target in certain malignancies including chondrosarcomas. In this study, we demonstrated that a nonsteroidal anti‐inflammatory drug, zaltoprofen, could induce PPAR *γ* activation and elicit anti‐tumor effects in chondrosarcoma cells. Zaltoprofen was found to induce expressions of PPAR *γ* mRNA and protein in human chondrosarcoma SW1353 and OUMS27 cells, and induce PPAR *γ*‐responsible promoter reporter activities. Inhibitory effects of zaltoprofen were observed on cell viability, proliferation, migration, and invasion, and the activity of matrix metalloproteinase‐2 (MMP2); these effects were dependent on PPAR *γ* activation and evidenced by silencing PPAR *γ*. Moreover, we showed a case of a patient with cervical chondrosarcoma (grade 2), who was treated with zaltoprofen and has been free from disease progression for more than 2 years. Histopathological findings revealed enhanced expression of PPAR *γ* and reduced expression of MMP2 after administration of zaltoprofen. These findings demonstrate that zaltoprofen could be a promising drug against the malignant phenotypes in chondrosarcomas via activation of PPAR *γ* and inhibition of MMP2 activity.

## Introduction

Chondrosarcomas are the second largest group of malignant bone tumors following osteosarcoma, and they arise from various sites including extremity, trunk, spine, and head 1. Because of their resistance to chemotherapy and radiotherapy, surgical resection is the only treatment for chondrosarcomas; however, they often recur after incomplete resection or metastasis 2. Therefore, novel strategies are crucial to overcome the malignancy of chondrosarcomas.

Peroxisome proliferator‐activated receptor gamma (PPAR*γ*) is a ligand‐activated transcription factor that belongs to the nuclear hormone receptor superfamily 3. PPAR*γ* plays a central role in the differentiation of adipocytes and is reported to be expressed in various malignant tumors 4. Anti‐tumorigenic effects through ligand activation of PPAR*γ* have been demonstrated in certain malignancies 5, 6. Furthermore, in chondrosarcoma, PPAR*γ* has been considered as a possible therapeutic target 7. We have recently reported a case of giant cell tumor of bone, in which complete necrosis and high expression of PPAR*γ* appeared in the resected specimen after zaltoprofen administration 8. Zaltoprofen is a propionic‐acid derivative of nonsteroidal anti‐inflammatory drugs (NSAIDs) 8. Previous studies have shown that several NSAIDs could serve as exogenous ligands for PPAR*γ* 9, 10, 11. We consider the possibility of anti‐tumorigenic effects by zaltoprofen via the activation of PPAR*γ* in musculoskeletal neoplasms.

Matrix metalloproteinase‐2 (MMP2) is a member of the MMP family and can degrade matrix collagen and basement membrane, which plays a critical role in tumor invasion and metastasis 12, 13. Overexpression of MMP2 has been detected in various types of cancer, including chondrosarcomas 14. Therefore, the inhibition of MMP2 is a potent strategy to suppress tumor progression in chondrosarcoma 14, 15. Recent studies report that the activation of PPAR*γ* inhibited MMP2 production and tumor cell invasion in several types of cancer, indicating that downregulation of MMP2 by PPAR*γ* could represent an anti‐tumorigenic procedure 16, 17.

The purpose of this study was to assess the anti‐tumor effects of zaltoprofen via regulation of MMP2 and PPAR*γ* activity, and examine whether zaltoprofen could be a novel therapeutic agent in human chondrosarcoma.

## Material and Methods

### Cells and reagents

Human chondrosarcoma cell lines, OUMS27, and SW1353 were purchased from Japanese Collection of Research Bioresources Cell Bank (JCRB Cell Bank, Tokyo, Japan) and American Type Culture Collection (ATCC, Manassas, VA), respectively. They were cultured in Dulbecco's modified Eagle's medium (DMEM) (Wako Pure Chemical Industries, Ltd., Osaka, Japan) supplemented with 10% fetal bovine serum (FBS), penicillin (100 U/mL) and streptomycin (100 *μ*g/mL) at 37°C. Zaltoprofen and ARP100, a selective MMP2 inhibitor, were purchased from Santa Cruz (Santa Cruz Biotechnology, Inc., Santa Cruz, CA) and dissolved in dimethyl sulfoxide (DMSO).

### Western blot analysis

OUMS27 and SW1353 cells treated with various concentrations of zaltoprofen for 24 h were lysed by RIPA buffer (150 mmol/L NaCl, 1% sodium deoxycholate, 1% Triton X‐100, 0.1% SDS, 10 mmol/L Tris, pH 7.2, 100 *μ*mol/L sodium orthovanadate, and 50 mmol/L sodium fluoride) containing protease cocktail inhibitors (Sigma‐Aldrich Co., Munich, Germany). The cell lysates (100 *μ*g) were boiled in SDS sample buffer, resolved by SDS‐PAGE (12.5%), run on an e‐PAGEL (ATTO Co., Tokyo, Japan), and then transferred onto PVDF membrane (Merck Millipore, Darmstadt, Germany). The membranes were blocked with Odyssey Blocking Buffer PBS (LI‐COR Inc., Lincoln, NE) for 1 h and then reacted with a mouse monoclonal anti‐PPAR*γ* antibody (Santa Cruz Biotechnology, Inc., Santa Cruz, CA) at 4°C for 24 h. After washing with PBS with Tween‐20, the membranes were reacted with IRDye 680 anti‐mouse IgG (LI‐COR Inc., Lincoln, NE) for 30 min, and immunoreacted bands were visualized using the Odyssey Infrared Imaging system (LI‐COR Inc., Lincoln, NE). The glyceraldehyde‐3‐phosphate dehydrogenase (GAPDH) protein was used as a housekeeping protein and detected with a monoclonal anti‐GAPDH antibody (Santa Cruz Biotechnology, Inc., Santa Cruz, CA).

### Quantitative real‐time reverse transcription‐polymerase chain reaction (qRT‐PCR)

After treatment with zaltoprofen for 24 h, total RNA extracted from OUMS27 and SW1353 cells using Ambion TRIzol reagent (Invitrogen, Carlsbad, CA) was quantified using a NanoDrop Lite spectrophotometer (Thermo Fisher Scientific Inc., Waltham, MA). The RNA sample (100 ng) was reverse‐transcribed into cDNA on a T100 Thermal Cycler (Bio‐Rad Laboratories, Inc., Hercules, CA) using AffinityScript cDNA Synthesis Kit (Agilent Technologies Inc., Santa Clara, CA) in accordance with the manufacturer's instructions. The qRT‐PCR was performed with 10 ng cDNA per well on a StepOne^™^ Real‐time PCR System (Applied Biosystems, Waltham, MA) using the QuantiTect SYBR Green PCR Kits (Qiagen, Hilden, Germany). *GAPDH* was used as an internal control. Primers used for qRT‐PCR were as follows: primer for *PPARγ* was purchased by Qiagen (QuantiTect Primer Assay, PPARG). *GAPDH* forward, 5′‐TGCACCA‐CCAACTGCTTAGC‐3′; reverse, 5′‐GGCATGGACTGTGGTCATGAG‐3′.

### PPAR*γ* reporter assay

PPAR*γ* activity was assessed using a luciferase reporter gene assay kit (INDIGO Biosciences, Inc., State College, PA), according to the manufacturer's instructions 18. Briefly, reporter cells were dispensed into wells and then immediately treated with zaltoprofen. Following 24 h incubation, treatment media were discarded and luciferase detection reagent was added. The intensity of light emission from each sample well was quantified using a plate‐reading luminometer (TriStar LB 941 Multimode Microplate Reader, Berthold Technologies, Bad Wildbad, Germany).

### *PPARγ* knockdown

SureSilencing short hairpin RNA (shRNA) plasmid for human *PPARγ* (Qiagen, Hilden, Germany) was used to knockdown *PPARγ* gene. *PPARγ*‐specific shRNA (shPPAR) or nontarget control shRNA (shNTC) plasmid were transfected in OUMS27 and SW1353 cells using Attractene transfection reagent (Qiagen, Hilden, Germany) according to the manufacturer's protocol 19.Transfected cells were selected by resistance to 600 *μ*g/mL neomycin (G418 solution, Roche Diagnostics, Indianapolis, IN) and the knockdown efficiency was confirmed by qRT‐PCR. Insert sequence of *PPARγ*‐specific shRNA is CCACGAGATCATTACACAAT, and nontarget control shRNA is GGAATCTCATTCGATGCATAC. Finally, we established PPAR*γ* knockdown cell lines (SW‐1353^shPPAR^ and OUMS‐27^shPPAR^) and nontarget control cell lines (SW‐1353^shNTC^ and OUMS‐27^shNTC^).

### Cell proliferation assay

The cell proliferation assay was performed using a Cell Counting Kit‐8 (Dojindo Laboratory, Mashiki‐machi, Japan). Briefly, 5 × 10^3^ cells/well were seeded in 96‐well plates. After 24 h incubation at 37°C, indicated concentrations of zaltoprofen were added to the medium, and after further 72 h of incubation, the medium was removed and a Cell Counting Kit‐8 reagent was added to the plates. After a final incubation at 37°C for 2 h, the absorbance was measured at 450 nm by a microplate reader (iMark Microplate Absorbance Reader, Bio‐Rad Laboratories, Inc., Hercules, CA). In addition, 5‐ethynyl‐2′‐deoxyuridine (EdU) proliferation assay was carried out using Click‐iT^®^ EdU Alexa Fluor^®^ 555 Imaging Kit (Invitrogen, Carlsbad, CA) according to the manufacturer's protocol 20. Briefly, OUMS27^shPPAR^, OUMS27^shNTC^, SW1353^shPPAR^, and SW1353^shNTC^ were seeded on slide chambers and incubated overnight. After treatment with zaltoprofen (400 *μ*mol/L) or DMSO for 24 h, cells were treated with EdU (10 mmol/L) for 1 h. Then, they were fixed with 4% paraformaldehyde (Wako Pure Chemical Industries, Ltd., Osaka, Japan), washed with 3% bovine serum albumin in PBS, and permeabilized with 0.5% Triton X‐100. Cells were then incubated with the Click‐iT reaction cocktail, followed by Hoechst 33342 (NucBlue Live ReadyProbes Reagent, Invitrogen, Carlsbad, CA), and were observed using fluorescence microscope (BZ‐9000, Keyence Co., Osaka, Japan). The number of EdU‐positive cells and Hoechst 33342‐positive cells was counted using Image J software according to the manufacturer's instructions 21.

### Cell migration assay

Cell migration was evaluated by a monolayer denudation assay as described previously 22. Briefly, OUMS27^shPPAR^, OUMS27^shNTC^, SW1353^shPPAR^, and SW1353^shNTC^ were seeded at a density of 1 × 10^6^ cells/well and grown to confluence in a 6‐well plate. Cells were then wounded by denuding a strip of the monolayer approximately 1 mm in width with a 200 *μ*L pipette tip. After washing cells with serum‐free DMEM, DMSO (0.1%), zaltoprofen (200 or 400 *μ*mol/L), a selective MMP2 inhibitor, and ARP100 (25 *μ*mol/L) were added to each well, and then cells were incubated for 30 h. The rate of wound closure was assessed in six separate fields using Image J software 23.

### Cell invasion assay

The invasive potential of OUMS27^shPPAR^, OUMS27^shNTC^, SW1353^shPPAR^, and SW1353^shNTC^ was assessed using Matrigel invasion chambers (24‐well, 8 *μ*m pore size) (BD Biosciences, San Diego, CA) 24. Cells were plated into the upper chamber at an initial density of 4 × 10^4^ cell/ml in 0.5 mL serum‐free DMEM, and 0.5 mL of DMEM supplemented with 10% FBS and fibronectin 500 *μ*g/mL was added to the bottom chambers as chemoattractant. After 24 h incubation in the presence of DMSO (0.1%), zaltoprofen (200 or 400 *μ*mol/L) and ARP100 (25 *μ*mol/L) into both the upper and bottom chambers, the noninvasive cells that remained at the top chambers were removed by scraping using a cotton swab, and the invasive cells at the bottom of the membranes were fixed with methanol. Cells were then washed twice with PBS and stained with 1% crystal violet for 30 min at RT. The invasive cells were counted in six separate fields using an optical microscope at 20× magnification 24.

### Gelatin zymography analysis

The expression of activated MMPs in conditioned medium was assayed by gelatin zymography 25. The cells (OUMS27^shPPAR^, OUMS27^shNTC^, SW1353^shPPAR^, and SW1353^shNTC^) were seeded at a density of 1 × 10^6^ cells/well and grown to 70–80% confluency in a 6‐well plate. Then the cells were cultured in serum‐free DMEM containing DMSO (0.1%) or zaltoprofen (200 or 400 *μ*mol/L) for 24 h. Afterward, the supernatants were collected and mixed with 5× SDS sample buffer without reducing agent or heating. The samples were loaded onto a Gelatin‐zymography Precast GEL (Cosmo Bio Co., Ltd., Tokyo, Japan) and subjected to electrophoresis. Following electrophoresis, the gel was washed twice with 2.5% Triton X‐100, 10 mmol/L Tris‐HCl (pH 8.0) for 30 min to remove SDS, and then incubated in 1 *μ*mol/L ZnCl2, 10 mmol/L CaCl2, 0.2 mol/L NaCl, and 50 mmol/L Tris‐HCl (pH 8.0) for 28 h at 37°C. Enzyme activity was visualized as negative staining with Coomassie Brilliant Blue R250 and gel was washed with destaining solution until clear bands were visible.

### A clinical case of chondrosarcoma with zaltoprofen administration

A 34‐year‐old man presented with grade 1 chondrosarcoma of cervical spine underwent tumor excision by his previous doctor. After 1 year from initial surgery, recurrent tumor was detected and excised by the same doctor, and the pathological diagnosis of the resected section was grade 2 chondrosarcoma. Although, adjuvant radiotherapy was conducted, the tumor had gradually enlarged. After 3 years from second surgery, he was referred to our hospital for severe pain and numbness of the left upper arm. There was bone destruction of the preserved vertebral arch and the tumor invaded the spinal canal, excluding the dura of cervical spine from anterolateral side of C5‐6. We performed radiotherapy and tumor excision of the posterior side of C2‐4. However, the tumor of C5‐6 was unresectable; hence, 240 mg/day of zaltoprofen was administered to the patient against his persistent pain and numbness on the neck and the left upper arm. For approximately 2 years since zaltoprofen administration, the tumor remained within mild growth for grade 2 chondrosarcoma; however, instability and anteflexion of the cervical spine proceeded. Therefore, the patient underwent tumor excision and posterior fixation of the cervical spine. At the final follow‐up, the patient was alive with disease without distant metastasis for more than 7 years after the initial diagnosis and 4 years after the administration of zaltoprofen (Fig. 5). The resection paraffin sections of two different treatment periods, the second surgery (preadministration specimen, Pre) and final surgery (postadministration specimen, Post), were used for hematoxylin‐Eosin (HE) staining and PPAR*γ* and MMP2 immunofluorescent staining with anti‐PPAR*γ* antibody (Santa Cruz Biotechnology, Inc., Santa Cruz, CA) and anti‐MMP2 antibody (Santa Cruz Biotechnology, Inc., Santa Cruz, CA), respectively, in combination with IRDye 680 anti‐mouse IgG (LI‐COR Inc., Lincoln, NE) as a secondary antibody. Nuclear staining was performed with 4′, 6‐diamidino‐2‐phenylindole (DAPI).

### Consent for use of human specimens

Declaration of Helsinki protocols were followed and the patient gave their written, informed consent, which was approved by the Ethics Committee of Kanazawa University.

### Statistical analysis

Each experiment was repeated independently at least three times. All data are presented as mean ± SEM. Student's *t*‐test and ANOVA using the Tukey‐Kramer post hoc test were used when mean differences were identified between the groups. All *P*‐values were two‐sided and *P*‐values of 0.05 or less were considered statistically significant. All statistical analyses were performed with EZR (Saitama Medical Center, Jichi Medical University, Saitama, Japan) 26, which is a graphical user interface for R (The R Foundation for Statistical Computing, Vienna, Austria).

## Results

### Effects of zaltoprofen on the induction and activation of PPAR*γ* in chondrosarcoma cells

**Figure 1:**
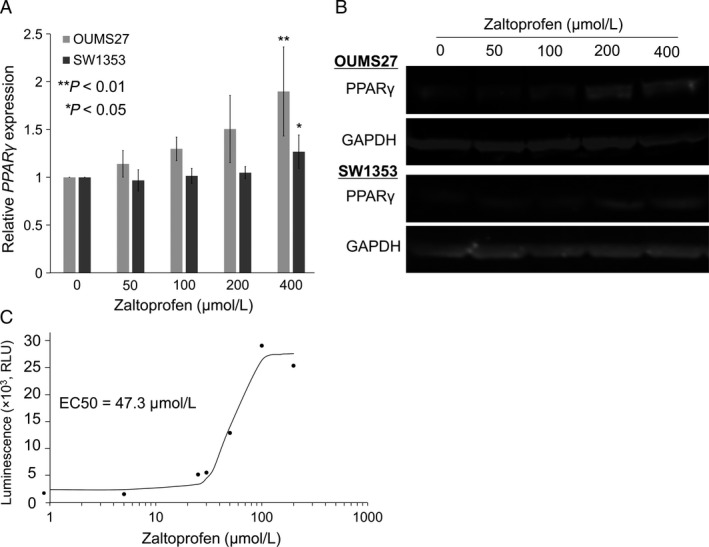
(A) Effects of zaltoprofen on *PPARγ* mRNA expression in OUMS27 and SW1353 cells by qRT‐PCR. Values represented as the mean ± SEM. Asterisks denote a statistically significant difference (*P <* *0.05 and **P <* *0.01) between cells with and without treatment of zaltoprofen (400 *μ*mol/L). (B) Effects of zaltoprofen on PPAR *γ* protein expression in OUMS27 and SW1353 cells by western blotting. GAPDH was used as an internal control. (C) PPAR *γ* activity was determined by luciferase reporter gene assay. Reporter cells were treated with zaltoprofen (0–200 *μ*mol/L) for 24 h, followed by measurement of luciferase activities.

qRT‐PCR demonstrated a significant upregulation of *PPARγ* mRNA in OUMS27 and SW1353 cells after 24 h‐treatment with 400 *μ*mol/L zaltoprofen (Fig. 1A). Moreover, zaltoprofen treatment dose‐dependently increased expression levels of PPAR*γ* protein in both cell lines as observed by western blotting (Fig. 1B), indicating that zaltoprofen induces *PPARγ* expression in chondrosarcoma cells. We next investigated the activity of PPAR*γ* using a luciferase reporter assay system. The zaltoprofen treatment could significantly and dose‐dependently upregulate PPAR*γ*‐responsible promoter activities, and the EC50 (effective concentration) was found to be 47.3 *μ*mol/L (Fig. 1C); this suggests a strong induction of PPAR*γ* activity, with a relatively mild upregulation of PPAR*γ* expression by zaltoprofen.

### Inhibitory effects of zaltoprofen on the viability and proliferation of chondrosarcoma cells

**Figure 2:**
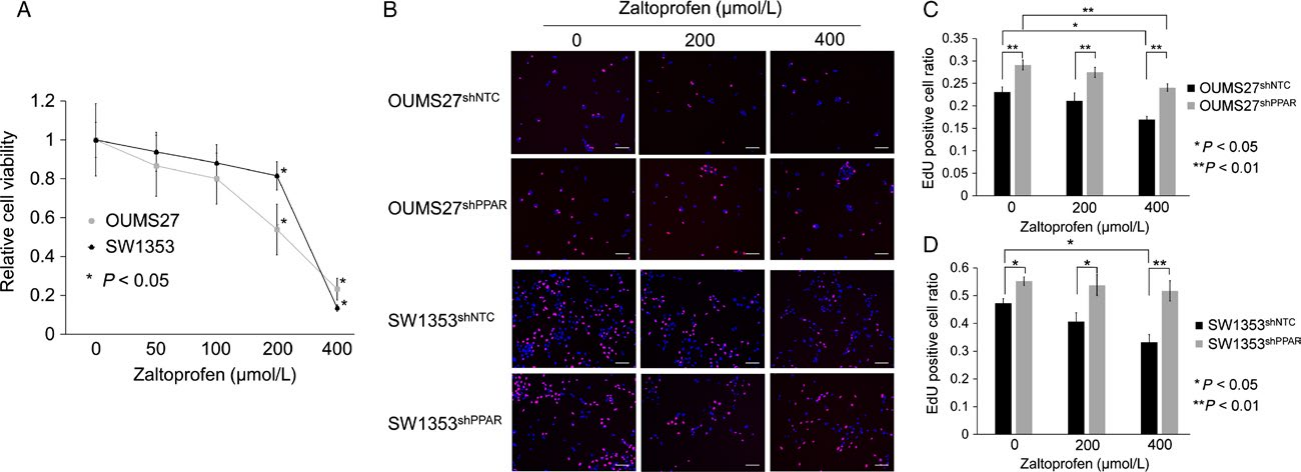
(A) Effects of zaltoprofen on the viability of OUMS27 and SW1353 cells. The cells were treated with or without zaltoprofen for 72 h, followed by WST assay. Asterisks denote a statistically significant difference (*P <* *0.05) between cells with and without treatment of zaltoprofen (200 or 400 *μ*mol/L). (B) Representative photos of 5‐ethynyl‐2′‐deoxyuridine (EdU) proliferation assay in PPAR *γ* knockdown cell lines, OUMS27^sh^ ^PPAR^ and SW1353^sh^ ^PPAR^, and the control cell lines, OUMS27^sh^ ^NTC^ and SW1353^sh^ ^NTC^ with or without the treatment of zaltoprofen for 24 h. Scale bar = 100 *μ*m. (C, D) The EdU‐positive cell ratio [EdU‐positive nuclei (red)/Hoechst 33342‐positive nuclei (blue)] in OUMS27 (C) and SW1353 cells (D). Values represented as the mean ± SEM. Asterisks denote a statistically significant difference (*P <* *0.05 and **P <* *0.01).

We next investigated whether zaltoprofen could reduce the viability of OUMS27 and SW1353 chondrosarcoma cell lines in vitro. When cells were treated with various concentrations (0–400 *μ*mol/L) of zaltoprofen for 72 h, cell viability of OUMS27 and SW1353 cells was significantly and dose‐dependently lowered by zaltoprofen treatment (Fig. 2A). In addition, cell proliferation assay was performed to evaluate EdU incorporation into the nucleus. The number of EdU‐incorporated cells per Hoechst 33342‐positive nuclei ratio was calculated and the involvement of PPAR*γ* in the cell proliferation was analyzed with or without downregulation of PPAR*γ* by a specific shRNA knockdown system (Fig. 2B). As a result, the EdU‐positive cell ratio was significantly and dose‐dependently decreased by the treatment of zaltoprofen in both OUMS27 and SW1353 cells, when compared to controls (Fig. 2C and D). Moreover, the downregulation of the EdU‐positive cell ratio by zaltoprofen was significantly inhibited by PPAR*γ* silencing in chondrosarcoma cells, OUMS27^shPPAR^ and SW1353^shPPAR^ cells (Fig. 2C and D). These data suggest that inhibitory effects on cell viability and proliferation by zaltoprofen were via PPAR*γ*.

### Inhibitory effects of zaltoprofen on cell migration and invasion in chondrosarcoma cells

**Figure 3:**
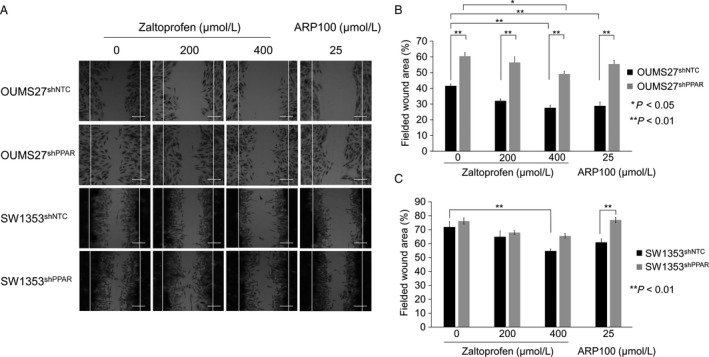
(A) Cell migration assay was carried out using PPAR *γ* knockdown cell lines, OUMS27^sh^ ^PPAR^ and SW1353^sh^ ^PPAR^, and the control cell lines, OUMS27^sh^ ^NTC^ and SW1353^sh^ ^NTC^ with or without the treatment of zaltoprofen. A selective inhibitor of MMP2, ARP100, was used at 25 *μ*mol/L. Scale bar = 100 *μ*m. (B, C) The fielded wound area (%) was calculated in OUMS27 (B) and SW1353 cells (C). Values represented as the mean ± SEM. Asterisks denote a statistically significant difference (*P <* *0.05 and **P <* *0.01).

**Figure 4:**
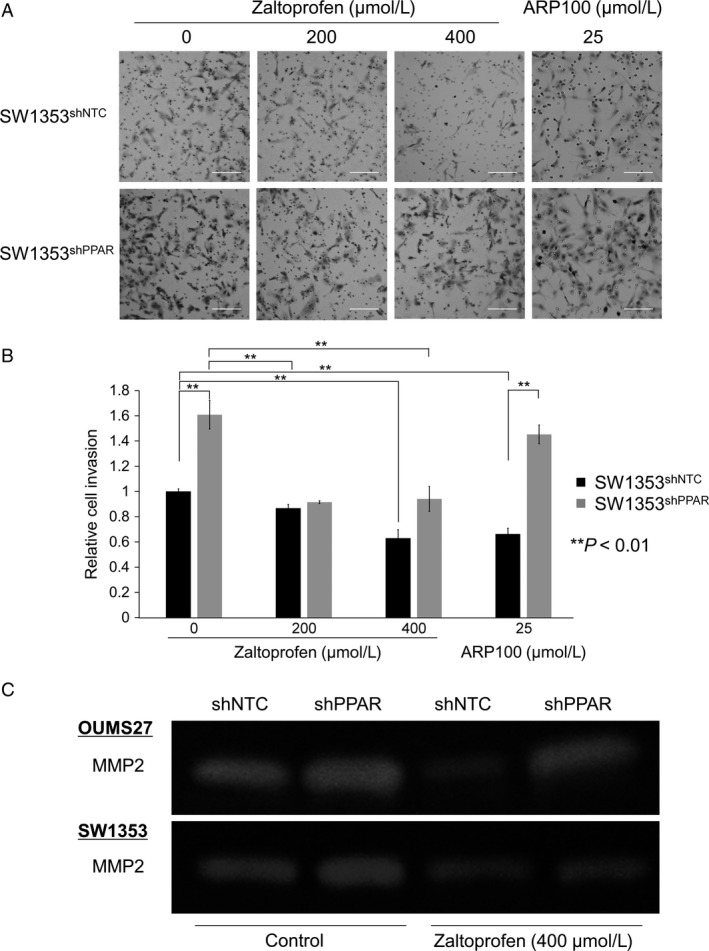
(A) Cell invasion assay was carried out using a PPAR *γ* knockdown cell line SW1353^sh^ ^PPAR^ and the control cell line SW1353^sh^ ^NTC^ with or without the treatment of zaltoprofen. A selective inhibitor of MMP2, ARP100, was used at 25 *μ*mol/L. Scale bar = 100 *μ*m. (B) Relative invaded cell number was calculated. Values represented as the mean ± SEM. Asterisks denote a statistically significant difference (**P <* *0.01). (C) Gelatin zymography. Effects of zaltoprofen (400 *μ*mol/L) on MMP2 activities in PPAR *γ* knockdown cell lines, OUMS27^sh^ ^PPAR^ and SW1353^sh^ ^PPAR^, and the control cell lines, OUMS27^sh^ ^NTC^ and SW1353^sh^ ^NTC^.

To examine whether zaltoprofen contributes to tumor malignancy in terms of metastatic potentials, we performed in vitro cell migration assays and cell invasion assay using transwell inserts coated with a reconstituted basement membrane barrier (Matrigel). First, in vitro cell migration assays were performed and fielded wound area (%) was calculated (Fig. 3). Zaltoprofen at a concentration of 400 *μ*mol/L and 200 *μ*mol/L significantly and partially inhibited cell migration of both OUMS27 and SW1353 cells, respectively (Fig. 3). Positive control ARP100 (25 *μ*mol/L), a selective MMP2 inhibitor, also showed a significant inhibitory effect on cell migration (Fig. 3). *PPARγ* silencing in chondrosarcoma cells, OUMS27^shPPAR^ and SW1353^shPPAR^, exhibited a significant inhibition of cell migration even in the nontreated control and following cancelation of the inhibitory effect by zaltoprofen, as well as ARP100 (Fig. 3). Second, we employed a Matrigel cell invasion assay with zaltoprofen and ARP100. Zaltoprofen significantly and dose‐dependently inhibited cell invasion of SW1353 cells (Fig. 4A and B). ARP100 significantly inhibited cell invasion of SW1353 cells at a concentration of 25 *μ*mol/L (Fig. 4A and B). In the absence of zaltoprofen or ARP100, *PPARγ* silencing SW1353^shPPAR^ cells showed a significant increase in cell invasion when compared to control SW1353^shNTC^ cells, suggesting a PPAR*γ*‐dependent inhibition of cell invasion, which was on the contrary to obtained data on cell migration (Fig. 4A and B). However, zaltoprofen treatment significantly blocked invasion of SW1353^shPPAR^ cells, speculating the involvement of a PPAR*γ*‐independent effect of zaltoprofen (Fig. 4A and B).

### Inhibitory effects of zaltoprofen on MMP2 expression in chondrosarcoma cells

The data on the selective MMP2 inhibitor, ARP100, led us to investigate the relationship between MMP2 and zaltoprofen. Gelatin zymography showed downregulation of MMP2 enzyme activities by zaltoprofen at 400 *μ*mol/L in OUMS27^shNTC^ and SW1353^shNTC^ cells (Fig. 4C). However, in OUMS27^shPPAR^ and SW1353^shPPAR^ cells, the downregulation of MMP2 activities by zaltoprofen was inhibited by *PPARγ* silencing (Fig. 4C), thus, suggesting that a zaltoprofen‐induced PPAR*γ* activation could reduce MMP2 activities in chondrosarcoma cells.

### A clinical case of chondrosarcoma treated with zaltoprofen

**Figure 5:**
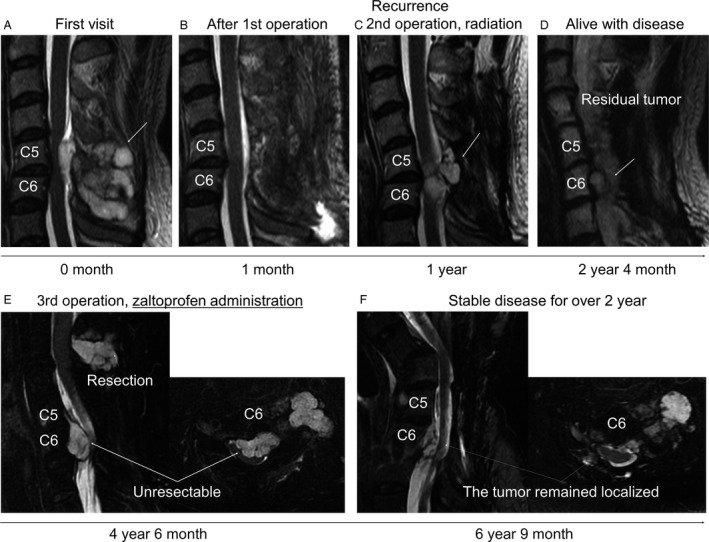
(A, B) Sagittal, MRI T2 weighted image of cervical spine at first visit on the initial hospital before (A) and after (B) the surgery. The tumor (arrow) was resected (1st operation), which markedly compressed the spinal cord. (C) Local recurrence was detected at C5‐6 and tumor excision (2nd operation) was then performed after 1 year from the initial diagnosis. An arrow indicates the tumor. (D) The residual tumor (arrow). (E) Sagittal, MRI T2 fat suppression image. The tumors markedly enlarged and severe pain occurred in his left hand. The tumor of C2‐4 was resected, while the tumor of C5‐6 was unresectable. Zaltoprofen (240 mg/day) was then administrated for the pain. (F) Before the final operation for severe cervical kyphosis, MRI T2 fat suppression image showed the localized tumor of C5‐C6 remained.

**Figure 6:**
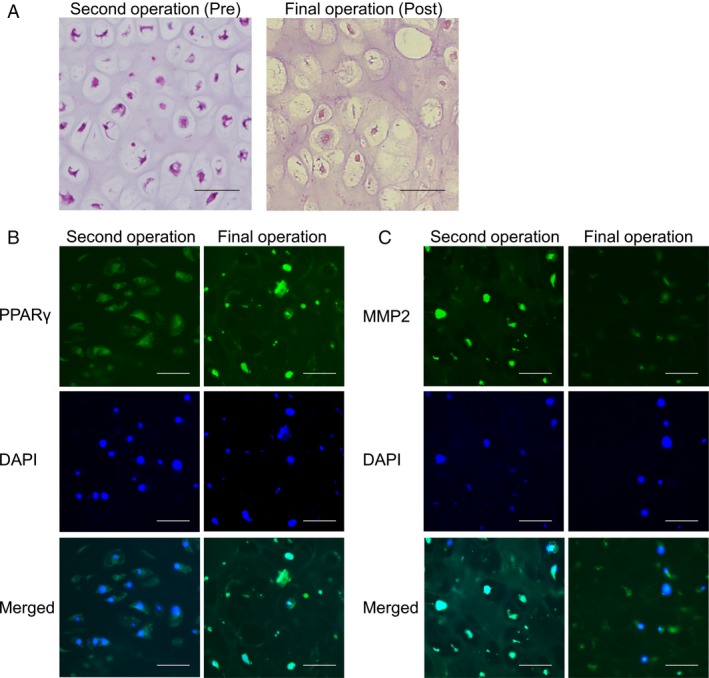
Histopathological findings of the tumors at the second operation (preadministration of zaltoprofen, Pre) and the final operation (postadministration of zaltoprofen, Post). Scale bar = 25 *μ*m. (A) HE staining. (B, C) Immunofluorescent stainings for PPAR *γ* (B) and MMP2 (C). DAPI, a nuclear staining.

We experienced a case of chondrosarcoma with unintentional zaltoprofen treatment. (Fig. 5). A specimen was obtained from the patient with grade 2 chondrosarcoma before administration of zaltoprofen (preadministration, Pre), and the microscopic data showed several nuclei with irregular shape and mitoses (Fig. 6A). In contrast, in the postadministration (Post) specimens, less nuclei with irregular shape or mitoses were found, and sporadic myxoid degeneration and necrotic portions were observed (Fig. 6A). Immunofluorescent staining of PPAR*γ* and MMP2 demonstrated that the expression of PPAR*γ* was enhanced, but that of MMP2 was reduced, after the administration of zaltoprofen (Fig. 6B and C).

## Discussion

In the present study, we found that zaltoprofen could dose‐dependently upregulate PPAR*γ* activities and significantly, but mildly, induce PPAR*γ* expression in human chondrosarcoma OUMS27 and SW1353 cells (Fig. 1). Zaltoprofen inhibited cell viability and proliferation as well as cell migration in a PPAR*γ*‐dependent manner in OUMS27 and SW1353 cells (Figs. 2 and 3). Although cell invasion was significantly reduced by zaltoprofen treatment, PPAR*γ*‐knockdown could not cancel the effects of zaltoprofen (Fig. 4A and B), suggesting two possibilities including an inhibitory action of PPAR*γ* against cell invasion and a PPAR*γ*‐independent effect of zaltoprofen on cell invasion. In addition, the downregulation of MMP2 activity by zaltoprofen was dependent on PPAR*γ* expression (Fig. 4C).

It has been reported that PPAR*γ* is closely associated with malignant tumors 4. Recent studies suggest that PPAR*γ* ligands can be useful anti‐tumor agents in cancer cells 5, 6. Endogenous and exogenous ligands for PPAR*γ* include fatty acids, eicosanoids, fibrates, thiazolidine derivatives, angiotensin receptor blockers, and NSAIDs 3, 8, 27. Among them, NSAIDs such as indomethacin, diclofenac, oxaprozin, and zaltoprofen have been reported as potent ligands for PPAR*γ* 8 and anti‐tumor agents against colorectal and lung cancers 28, 29. This is the first report of anti‐cancer effects of zaltoprofen against malignant musculoskeletal tumors. Activation of PPAR*γ* is reported to drive the positive feedback loop upregulating the *PPARG* gene *per se* via direct binding of PPAR*γ*/RXR*α* heterodimers to the *PPARG* promoter 30. However, the involvement of other transcriptional activation factors for *PPARG*, including CEBPs and KLFs, is still unclear in the PPAR*γ* upregulation by zaltoprofen. It is also interesting to examine whether zaltoprofen can selectively activate not only PPAR*γ*, but also PPAR*α* or PPAR*δ*, in terms of the anti‐tumor effects of zaltoprofen. Further studies are required to elucidate the overall mechanisms of action of zaltoprofen on the anti‐cancer activity.

MMP2, the 72 kDa gelatinase A/Type IV collagenase, plays an important role in the tumorigenic processes, such as tumor invasion and metastasis 12, 31. Therefore, the inhibition of MMP2 activation has been suggested to be a potent strategy for the prevention of cancer cell metastasis 31. To date, several MMP2 inhibitors have been developed for clinical use. In chondrosarcomas, the expression of MMP2 has been confirmed and demonstrated a significant correlation with the tumor histological grade linking to the prognosis 14. Therefore, MMP2 could be a possible therapeutic target to reduce local spread and invasion in chondrosarcomas. Lai et al. reported that alendronate, a second‐generation bisphosphonate, inhibited MMP2 production and the invasion of a chondrosarcoma cell line 15. Recent studies have also demonstrated that PPAR*γ* activation could inhibit cell migration and invasion through the downregulation of MMP2 in cancer cells 16, 17. In this study, we first discovered the downregulation of MMP2 activity in a PPAR*γ*‐dependent fashion by zaltoprofen.

We experienced a case of chondrosarcoma with unintentional zaltoprofen treatment. (Fig. 5). It is known that grade 2 chondrosarcoma progresses destructively and metastasizes. Local recurrence and metastasis rates have been reported to be 27% and 50%, respectively, in grade 2 chondrosarcoma, and 70% of chondrosarcoma cases with local recurrence have developed metastasis 32. Interestingly, for approximately 2 years during administration of zaltoprofen, the tumor remained localized without distant metastasis even though the tumor was grade 2 chondrosarcoma. Histopathological findings showed upregulation of PPAR*γ* and downregulation of MMP2 after the administration of zaltoprofen in the patient (Fig. 6). We believe that a long‐term administration of zaltoprofen could reduce the local spreading and metastatic abilities of the tumor cells.

In conclusion, this study showed that zaltoprofen activated PPAR*γ* and subsequently decreased MMP2 activity in human chondrosarcoma cells, thereby contributing to anti‐tumor effects against cell viability, proliferation, migration, and invasion. A beneficial effect of zaltoprofen was also demonstrated in a patient with grade 2 chondrosarcoma. Therefore, these findings suggest that zaltoprofen could be a novel promising remedy against chondrosarcomas via PPAR*γ* activation and MMP2 inhibition. Future studies using orthotopic xenograft mouse models will be required to show the in vivo effect of zaltoprofen on chondrosarcomas, based on previous reports of osteosarcoma 33, 34, rhabdomyosarcoma 35, and undifferentiated pleomorphic sarcoma 36, 37, 38 with various anti‐cancer drugs. In addition, large prospective cohort studies will be required to draw a final conclusion about the efficacy of zaltoprofen and to evaluate the effect of clinical applications in patients with chondrosarcoma.

## Conflict of Interest

None declared.

## References

[1] Dorfman, H. D. , and B. Czerniak . 1995 Bone cancers. Cancer 75:203–210. 8000997 10.1002/1097-0142(19950101)75:1+<203::aid-cncr2820751308>3.0.co;2-v

[2] Lee, F. Y. , H. J. Mankin , G. Fondren , M. C. Gebhardt , D. S. Springfield , A. E. Rosenberg , et al. 1999 Chondrosarcoma of bone: an assessment of outcome. J. Bone Joint Surg. Am. 81A:326–338. 10.2106/00004623-199903000-00004 10199270

[3] Kersten, S. , B. Desvergne , and W. Wahli . 2000 Roles of PPARs in health and disease. Nature 405:421–424. 10839530 10.1038/35013000

[4] Koeffler, H. P. 2003 Peroxisome proliferator‐activated receptor gamma and cancers. Clin. Cancer Res. 9:1–9. 12538445

[5] Fröhlich, E. , and R. Wahl . 2015 Chemotherapy and chemoprevention by thiazolidinediones. Biomed. Res. Int. 2015:845340. 25866814 10.1155/2015/845340 PMC4383438

[6] Robbins, G. T. , and D. Nie . 2012 PPAR gamma, bioactive lipids, and cancer progression. Front. Biosci. (Landmark Ed.) 17:1816–1834. 22201838 10.2741/4021 PMC3409468

[7] Nishida, K. , T. Kunisada , Z. N. Shen , Y. Kadota , K. Hashizume , and T. Ozaki . 2008 Chondrosarcoma and peroxisome proliferator‐activated receptor. PPAR Res. 2008:250568. 18725985 10.1155/2008/250568 PMC2517661

[8] Takeuchi, A. , N. Yamamoto , H. Nishida , H. Kimura , H. Ikeda , and H. Tsuchiya . 2013 Complete necrosis of a giant cell tumor with high expression of PPAR*γ*: a case report. Anticancer Res. 33:2169–2174. 23645771

[9] Yamazaki, R. , N. Kusunoki , T. Matsuzaki , S. Hashimoto , and S. Kawai . 2002 Nonsteroidal anti‐inflammatory drugs induce apoptosis in association with activation of peroxisome proliferator‐activated receptor gamma in rheumatoid synovial cells. J. Pharmacol. Exp. Ther. 302:18–25. 12065695 10.1124/jpet.302.1.18

[10] Knopfová, L. , and J. Smarda . 2010 The use of Cox‐2 and PPAR*γ* signaling in anti‐cancer therapies. Exp. Ther. Med. 1:257–264. 22993537 10.3892/etm_00000040 PMC3445927

[11] Ghanqhas, P. , S. Jain , C. Rana , and S. N. Sanyal . 2016 Chemopreventive action of non‐steroidal anti‐inflammatory drugs on the inflammatory pathways in colon cancer. Biomed. Pharmacother. 78:239–247. 26898448 10.1016/j.biopha.2016.01.024

[12] Egeblad, M. , and Z. Werb . 2002 New functions for the matrix metalloproteinases in cancer progression. Nat. Rev. Cancer 2:161–174. 11990853 10.1038/nrc745

[13] Bjorklund, M. , and E. Koivunen . 2005 Gelatinase‐mediated migration and invasion of cancer cells. Biochim. Biophys. Acta 1755:37–69. 15907591 10.1016/j.bbcan.2005.03.001

[14] Sakamoto, A. , Y. Oda , Y. Iwamoto , and M. Tsuneyoshi . 1999 Expression of membrane type 1 matrix metalloproteinase, matrix metalloproteinase 2 and tissue inhibitor of metalloproteinase 2 in human cartilaginous tumors with special emphasis on mesenchymal and dedifferentiated chondrosarcoma. J. Cancer Res. Clin. Oncol. 125:541–548. 10473866 10.1007/s004320050314 PMC12169196

[15] Lai, T. J. , S. F. Hsu , T. M. Li , H. C. Hsu , J. G. Lin , C. J. Hsu , et al. 2007 Alendronate inhibits cell invasion and MMP‐2 secretion in human chondrosarcoma cell line. Acta Pharmacol. Sin. 28:1231–1235. 17640487 10.1111/j.1745-7254.2007.00607.x

[16] Li, Y. , D. W. Zhang , D. Q. Lin , and L. Q. Cao . 2015 Peroxisome proliferator‐activated receptor‐*γ* inhibits pancreatic cancer cell invasion and metastasis via regulating MMP‐2 expression through PTEN. Mol. Med. Rep. 12:6255–6260. 26299428 10.3892/mmr.2015.4224

[17] Chuang, C. H. , C. L. Yeh , S. L. Yeh , E. S. Lin , L. Y. Wang , and Y. H. Wang . 2016 Quercetin metabolites inhibit MMP‐2 expression in A549 lung cancer cells by PPAR‐*γ* associated mechanisms. J. Nutr. Biochem. 33:45–53. 27260467 10.1016/j.jnutbio.2016.03.011

[18] Shah, B. S. , M. Chen , T. Suzuki , M. Embree , K. Kong , C. H. Lee , et al. 2017 Pyrintegrin induces soft tissue formation by transplanted or endogenous cells. Sci. Rep. 27:36402. 10.1038/srep36402 PMC5269584 28128224

[19] Noutsios, G. T. , P. Silveyra , F. Bhatti , and J. Floros . 2013 Exon B of human surfactant protein A2 mRNA, alone or within its surrounding sequences, interacts with 14‐3‐3; role of cis‐elements and secondary structure. Am. J. Physiol. Lung Cell. Mol. Physiol. 304:722–735. 10.1152/ajplung.00324.2012 PMC3680765 23525782

[20] Peters, A. A. , P. T. Simpson , J. J. Bassett , J. M. Lee , L. Da Silva , L. E. Reid , et al. 2012 Calcium channel TRPV6 as a potential therapeutic target in estrogen receptor‐negative breast cancer. Mol. Cancer Ther. 11:2158–2168. 22807578 10.1158/1535-7163.MCT-11-0965

[21] Grishagin, I. V. 2015 Automatic cell counting with Image. J. Anal. Biochem. 473:63–65. 10.1016/j.ab.2014.12.007 25542972

[22] Yarrow, J. C. , Z. E. Perlman , N. J. Westwood , and T. J. Mitchison . 2004 A high‐throughput cell migration assay using scratch wound healing, a comparison of image based readout methods. BMC Biotechnol. 4:21. 15357872 10.1186/1472-6750-4-21 PMC521074

[23] Silva Nunes, J. P. , and A. A. Dias . 2017 ImageJ macros for the user‐friendly analysis of soft‐agar and wound‐healing assays. Biotechniques 62:175–179. 28403808 10.2144/000114535

[24] Takeuchi, A. , Y. Yamamoto , S. Munesue , A. Harashima , T. Watanabe , H. Yonekura , et al. 2013 Low molecular weight heparin suppresses receptor for advanced glycation end products‐mediated expression of malignant phenotype in human fibrosarcoma cells. Cancer Sci. 104:740–749. 23421467 10.1111/cas.12133 PMC7657155

[25] Sato, H. , T. Takino , Y. Okada , J. Cao , A. Shinagawa , E. Yamamoto , et al. 1994 A matrix metalloproteinase expressed on the surface of invasive tumour cells. Nature 370:61–65. 8015608 10.1038/370061a0

[26] Kanda, Y. 2013 Investigation of the freely‐available easy‐to‐use software “EZR” (Easy R) for medical statistics. Bone Marrow Transplant. 48:452–458. 23208313 10.1038/bmt.2012.244 PMC3590441

[27] Kroker, A. J. , and J. B. Bruning . 2015 Review of the structural and dynamic mechanisms of PPAR*γ* partial agonism. PPAR Res. 2015:816856. 26435709 10.1155/2015/816856 PMC4578752

[28] Moon, C. M. , J. H. Kwon , J. S. Kim , S. H. Oh , K. Jin Lee , J. J. Park , et al. 2014 Nonsteroidal anti‐inflammatory drugs suppress cancer stem cells via inhibiting PTGS2 (cyclooxygenase 2) and NOTCH/HES1 and activating PPARG in colorectal cancer. Int. J. Cancer 134:519–529. 23852449 10.1002/ijc.28381

[29] Kato, T. , H. Fujino , S. Oyama , T. Kawashima , and T. Murayama . 2011 Indomethacin induces cellular morphological change and migration via epithelial‐mesenchymal transition in A549 human lung cancer cells: a novel cyclooxygenase‐inhibition‐independent effect. Biochem. Pharmacol. 82:1781–1791. 21840302 10.1016/j.bcp.2011.07.096

[30] Wakabayashi, K. , M. Okamura , S. Tsutsumi , N. S. Nishikawa , T. Tanaka , I. Sakakibara , et al. 2009 The peroxisome proliferator‐activated receptor gamma/retinoid X receptor alpha heterodimer targets the histone modification enzyme PR‐Set7/Setd8 gene and regulates adipogenesis through a positive feedback loop. Mol. Cell. Biol. 29:3544–3555. 19414603 10.1128/MCB.01856-08 PMC2698772

[31] Coussens, L. M. , B. Fingleton , and L. M. Matrisian . 2002 Matrix metalloproteinase inhibitors and cancer: trials and tribulations. Science 295:2387–2392. 11923519 10.1126/science.1067100

[32] Fiorenza, F. , A. Abudu , R. J. Grimer , S. R. Carter , R. M. Tillman , K. Ayoub , et al. 2002 Risk factors for survival and local control in chondrosarcoma of bone. J. Bone Joint Surg. Br. 84B:93–99. 10.1302/0301-620x.84b1.11942 11837841

[33] Igarashi, K. , K. Kawaguchi , T. Murakami , T. Kiyuna , K. Miyake , S. D. Nelson , et al. 2017 Intra‐arterial administration of tumor‐targeting Salmonella typhimurium A1‐R regresses a cisplatin‐resistant relapsed osteosarcoma in a patient‐derived orthotopic xenograft (PDOX) mouse model. Cell Cycle 16:1164–1170. 28494180 10.1080/15384101.2017.1317417 PMC5499917

[34] Igarashi, K. , T. Murakami , K. Kawaguchi , T. Kiyuna , K. Miyake , Y. Zhang , et al. 2017 A patient‐derived orthotopic xenograft (PDOX) mouse model of a cisplatinum‐resistant osteosarcoma lung metastasis that was sensitive to temozolomide and trabectedin: implications for precision oncology. Oncotarget 8:62111–62119. 28977930 10.18632/oncotarget.19095 PMC5617490

[35] Igarashi, K. , K. Kawaguchi , T. Kiyuna , T. Murakami , S. Miwa , S. D. Nelson , et al. 2017 Patient‐derived orthotopic xenograft (PDOX) mouse model of adult rhabdomyosarcoma invades and recurs after resection in contrast to the subcutaneous ectopic model. Cell Cycle 16:91–94. 27830986 10.1080/15384101.2016.1252885 PMC5270546

[36] Igarashi, K. , K. Kawaguchi , T. Murakami , T. Kiyuna , K. Miyake , N. Yamamoto , et al. 2017 A novel anionic‐phosphate‐platinum complex effectively targets an undifferentiated pleomorphic sarcoma better than cisplatinum and doxorubicin in a patient‐derived orthotopic xenograft (PDOX). Oncotarget 8:63353–63359. 28968995 10.18632/oncotarget.18806 PMC5609927

[37] Igarashi, K. , K. Kawaguchi , T. Murakami , T. Kiyuna , K. Miyake , A. S. Singh , et al. 2017 High efficacy of pazopanib on an undifferentiated spindle‐cell sarcoma resistant to first‐line therapy is identified with a patient‐derived orthotopic xenograft (PDOX) nude mouse model. J. Cell. Biochem. 118:2739–2743. 28176365 10.1002/jcb.25923

[38] Igarashi, K. , K. Kawaguchi , T. Kiyuna , T. Murakami , S. Miwa , S. D. Nelson , et al. 2017 Temozolomide combined with irinotecan caused regression in an adult pleomorphic rhabdomyosarcoma patient‐derived orthotopic xenograft (PDOX) nude‐mouse model. Oncotarget 8:75874–75880. 29100276 10.18632/oncotarget.16548 PMC5652670

